# Impact of Home Mobile Phone-Based Telemonitoring in Preventing Exacerbations and Hospitalizations Among Patients with Chronic Obstructive Pulmonary Disease: An IMTEC Study

**DOI:** 10.3390/jcm13216319

**Published:** 2024-10-23

**Authors:** Rania Kaddoussi, Khaoula Bel Haj Ali, Ekram Hajji, Houda Ben Soltane, Ikram Chamtouri, Randa Dhaoui, Salma Younes, Nesrine Fahem, Meriem Khalifa, Wafa Dhouib, Mohamed Amine Msolly, Adel Sekma, Hamdi Boubaker, Wahid Bouida, Semir Nouira

**Affiliations:** 1 Department of Pneumology, Fattouma Bourguiba Hospital, Monastir 5000, Tunisia; nesrinefahem@yahoo.fr; 2 Emergency Department and Research Laboratory LR12SP18, Fattouma Bourguiba University Hospital, Monastir 5000, Tunisia; belhajalikhaoula@yahoo.fr (K.B.H.A.); drrandadhaoui@yahoo.com (R.D.); hatemneb81@gmail.com (M.K.); m.amine.msolli@gmail.com (M.A.M.); adelsekma@hotmail.fr (A.S.); hamdiboubaker@hotmail.fr (H.B.); wahid.bouida@rns.tn (W.B.); semirnouiraurg@gmail.com (S.N.); 3 Department of Endocrinology, Fattouma Bourguiba Hospital, Monastir 5000, Tunisia; ekramhajji7@gmail.com; 4 Department of Emergency, Farhat Hached Hospital, Sousse 4021, Tunisia; bensoltanehouda@yahoo.fr (H.B.S.); cabinetdrchakroun@gmail.com (S.Y.); 5 Department of Cardiology, Fattouma Bourguiba Hospital, Monastir 5000, Tunisia; ikram_chamtouri@hotmail.fr; 6 Department of Community Health Service, Fattouma Bourguiba Hospital, Monastir 5000, Tunisia; wafa.dhouib20@yahoo.fr

**Keywords:** COPD, telemedicine, exacerbation, hospitalization

## Abstract

COPD is a major public health problem due to its high morbidity and mortality. The evolution of COPD is marked by the occurrence of acute exacerbations (AECOPD). One of the major causes of AECOPD is non-adherence treatment. Telemedicine is an accessible educational tool that can help physicians to provide continuous, accessible educational support and monitoring for patients with COPD. **Objectives**: This study aimed to determine the impact of therapeutic education via phone-based telemedicine on ED visits and/or hospitalizations for AECOPD. **Methods**: This is a randomized controlled trial carried out in the emergency department (ED) of Fattouma Bourguiba Monastir over a period of 7 consecutive months, including patients admitted with a final diagnosis of AECOPD. Patients were randomly assigned to receive standard care (STD) or weekly phone-based telemonitoring (TLM). Outcomes (exacerbation and the need for hospitalization for AECOPD) were assessed at a 1-, 3-, and 6-month follow-up after being discharged from the hospital. **Results**: We included 163 patients (57 patients in the TLM group and 106 patients in the STD group). The mean age of the study population was 66.5 ± 12.5 years. The TLM group exhibited a significantly lower risk of ED visits for AECOPD compared to the STD group, with an odds ratio of 0.13 (95% CI: 0.04–0.40) and a *p*-value ≤ 0.001. Additionally, the TLM group had a notably lower hospitalization rate for AECOPD compared to the control group (15.8% vs. 44.3%, respectively), with an odds ratio of 0.23 (95% CI: 0.10–0.52) and a *p*-value < 0.001. The all-cause death rate was also lower in the TLM group at the 6-month follow-up. **Conclusions**: Telemedicine represents an innovative approach that could improve the management of patients with COPD.

## 1. Introduction

Chronic obstructive pulmonary disease (COPD) is a chronic respiratory condition that triggers permanent and progressive airway blockage [1]. COPD is a serious public health concern with substantial morbidity and mortality [2]. According to the World Health Organization (WHO), COPD was the third most common cause of death in 2021 [3]. Despite its high prevalence, COPD remains misdiagnosed among two-thirds of cases [3]. The natural history of COPD is marked with acute exacerbation (EACOPD), which accelerates the evolution of the disease and the deterioration of respiratory function, impacting both patients’ quality of life and their survival [4]. Poor treatment adherence is responsible for 23.6% to 80% of EACOPD cases [5,6]. Therefore, pharmacological treatment must be routinely paired with non-pharmacological strategies which can prevent EACOPD, slow disease progression, and improve prognosis. Among these strategies, therapeutic education and self-management plans are important components of COPD management [7].

Therapeutic education typically occurs during consultations or hospitalizations. However, the effectiveness of these educational sessions is often constrained by time limitations during the consultations and patients’ short-term memory challenges. Consequently, there is a need for more accessible educational tools. Telemedicine has emerged as a valuable solution, particularly since the onset of the COVID-19 pandemic, providing a means to deliver continuous, accessible educational support [8]. Thus, our study aimed to determine the impact of home mobile phone-based telemonitoring in preventing emergency department (ED) consultation for exacerbations and hospitalizations of patients with AECOPD.

## 2. Materials and Methods

### 2.1. The Study Design

A randomized controlled trial was carried out in the emergency department (ED) at the Fattouma Bourguiba University Hospital of Monastir during a period of 7 months, and a parallel format was followed (i.e., from August 2023 to February 2024).

### 2.2. Study Population

Patients aged more than 18 years old with confirmed diagnosis of group E COPD (chronic obstructive pulmonary disease) [1] who consulted the ED for AECOPD were included. Diagnosis of COPD was confirmed by a spirometry using the GOLD (Global Initiative for Chronic Obstructive Lung Disease) 2023 criteria [1], indicating a non-reversible ventilatory deficit (FEV 1/FVC < 0.70 following bronchodilation, i.e., reduced forced expiratory volume in one second (FEV1) and forced vital capacity (FVC)) [1].

EACOPD is defined as an event characterized by increased dyspnea and/or cough and sputum that worsens in <14 days, which may be accompanied by tachypnea and/or tachycardia, and is often associated with increased local and systemic inflammation caused by infection, pollution, or other insult to the airways [1]. The non-inclusion criteria were other etiologies of dyspnea (e.g., asthma, cancer, fibrosis, apnea syndrome), pregnant women given the added risk of exacerbation due to pregnancy, patients with dementia given the possibility of forgetting treatment due to their dementia, which may represent a risk factor for exacerbation, or any other cause that may prevent remote monitoring from being set up, and patients participating concurrently in another trial involving a therapeutic or non-therapeutic intervention that will interfere with the primary and secondary endpoints of this study. Patients who did not attend all control sessions or did not respond to the phone calls were excluded from the final analysis.

### 2.3. Study Protocol

Participants were recruited directly from ED from August 2023 to September 2023. Participants were included before being discharged from the hospital following an EABPCO after verifying their eligibility criteria. Potentially eligible patients were contacted by a co-investigator, who explained the purpose of the study, provided them with an information sheet. and emphasized the confidential and anonymous nature of the study. Patients showing interest in taking part in the study were included. An informed written consent was signed by all participants before inclusion. Patients were then randomly assigned (1:2) using a computer-generated allocation performed via the Internet (http://www.randomized.org) (accessed on 1 September 2023) by an independent investigator to one of the two study groups.

1. Standard care group (STD): patients will receive usual follow-up via a pulmonology consultation at 1, 3, and 6 months after hospital discharge.
2. Telemonitoring group (TLM): patients included in this group will be monitored via weekly telephone calls by the same investigator experienced in COPD and telehealth for 1 month after hospital discharge, and thereafter they have a programmed consultation at 1, 3, and 6 months of follow-up.

The following data were collected using a questionnaire before discharge by a blinded investigator who had no access to the randomization sequence: Demographic data (e.g., age, sex, smoking status, occupation, telephone number, and address), medical history, and treatments used. The second part of the questionnaire was reserved to assess dyspnea level using the mMRC (modified medical research council) scale [2]. Questions were proposed in the Arabic dialect. The mMRC scale, a self-rating scale, measures the handicap caused by breathlessness in daily activities, with levels going from 0 to 4 [2]. Before discharge, all patients received optimal inhaled treatment under the supervision of the attending physician.

For the TLM group, telephone calls lasted 15 to 20 min and focused on the various axes of therapeutic education for a patient with COPD [1], including the characteristics of the disease, the treatments used, inhalation techniques, reminding patients when to take medication, inhalers, and rehabilitation exercises, the benefits of stopping active and passive smoking, the importance of practicing sport, the benefits of vaccination against pneumococcus, self-recognizing of early signs of exacerbation, and a personalized action plan. This content was standardized for all patients and conducted by the same independent investigator. For the STD group, patients were followed according to the usual schedule, which included a planned medical visit to the pulmonology consultation at 1, 3, and 6-months after hospital discharge. Patients received standard therapeutic education during the consultation.

### 2.4. Outcomes

#### 2.4.1. Primary Outcome

Data including ED visits and/or hospitalizations for AECOPD during the study period were collected at each time point.

#### 2.4.2. Secondary Outcome

Secondary outcomes included time to first hospital admission caused by exacerbation in COPD And rate of all-cause death.

### 2.5. Statical Analyses

The sample size was calculated based on data from another clinical trial which showed that the hazard ratio (HR) of COPD exacerbation was 0.66 (3). According to the specific formulas we obtain the following:

$$n=\frac{{\left(Z_{\frac{\alpha }{2}}+Z_{\beta }\right)}^{2}\;*\;(1+r)}{{\left[log(HR)\right]}^{2}\;*\;p(1-p)}$$

$$N=\frac{n}{EER}$$

The variables are described as follows:

N = total sample size (total number of participants);
n = number of events require;
r is the ratio of participants in the experimental group to the control group;
Zα/2 = 1.96 (for a 5% significance level);
Zβ = 0.84 (for 80% power);
Hazard ratio HR = 0.66;
Proportion of events *p* = 0.45 (control group);
EER = expected event rate.

And at α = 5% and power 1 − β = 80%, the total number of 163 patients was necessary (57 in the TLM group and 106 in the STD group). The unequal distribution of patients between the two groups is due to the difficulty of recruiting patients in the intervention group. Quantitative data were expressed as means ± standard deviation (SD), and categorical data as percentages. The chi-square test and Fisher’s exact test were appropriately used for categorical variables and percentage comparisons. The Student’s *t*-test was utilized to compare the means of quantitative variables. The adjustment of the odds ratios for sex was performed using binary logistic regression. The multivariate analysis was conducted using binary logistic regression with a stepwise descending approach. Adjustments were made for variables that displayed statistically significant differences in the univariate analysis to mitigate potential bias in the results. Robustness analyses were carried out by including and excluding subgroups and employing various models adjusted for the variable of interest (education level) to illustrate the final results. Values were considered significant when *p* was ≤0.05.

## 3. Results

**Figure 1:**
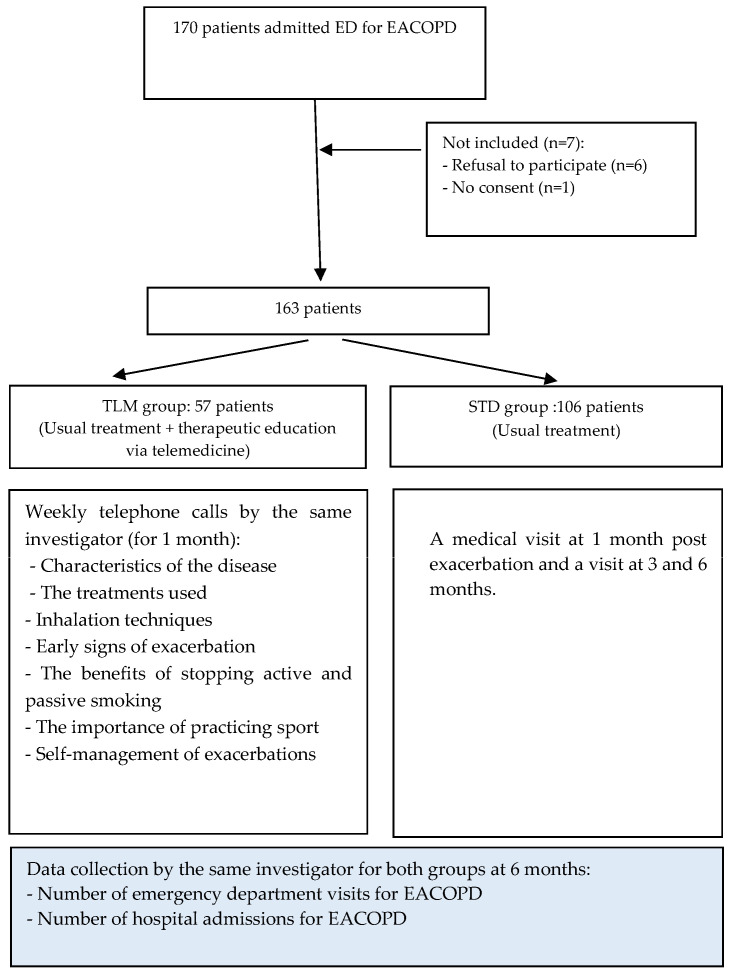
Flow chart of the study. COPD: chronic obstructive pulmonary disease; EA: acute exacerbation Abbreviations: STD: standard care, group (STD); TLM: telemonitoring group.

A total of 163 patients were included, 57 patients in the TLM group and 106 patients in the STD group (Figure 1).

The mean age was 66.5 years old ± 12.4. Hypertension was the most common comorbidity in both groups (63.2%, *p* = 0.99). The two study groups were statistically comparable in terms of socio-demographic and clinical characteristics as well as the mMRC stage 

A higher percentage of patients living more than 10 km away from the hospital was noted in the TLM group (39.6%) compared to the STD group (24.6%), with no statistically significant difference (Table 1).

**Figure 2:**
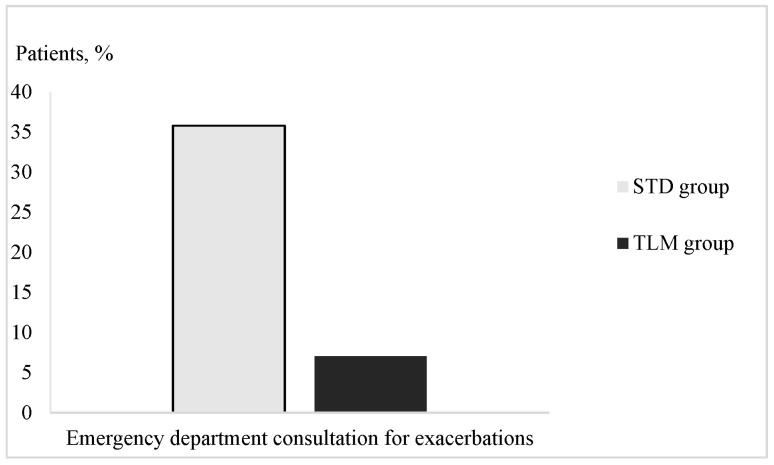
Emergency department consultation for exacerbations, rate among the study groups. Abbreviations: STD: standard care group (STD); TLM: telemonitoring group.

The TLM group had a significantly lower risk of ED visits for AECOPD compared to the STD group (7 vs. 35.8%, respectively), with an odds ratio of 0.13 (95% CI: 0.04–0.40) and a *p*-value ≤ 0.001 (Figure 2).

The TLM group also had a significantly lower rate of hospitalization for AECOPD compared to the STD group (15.8% vs. 44.3%, respectively), with an odds ratio of 0.23 (95% CI: 0.10–0.52) and a *p*-value < 0.001. All-cause death rate was lower in the TLM group compared to the STD group (5.7 vs. 1.8%; *p* = 0.001) (Table 2).

**Figure 3:**
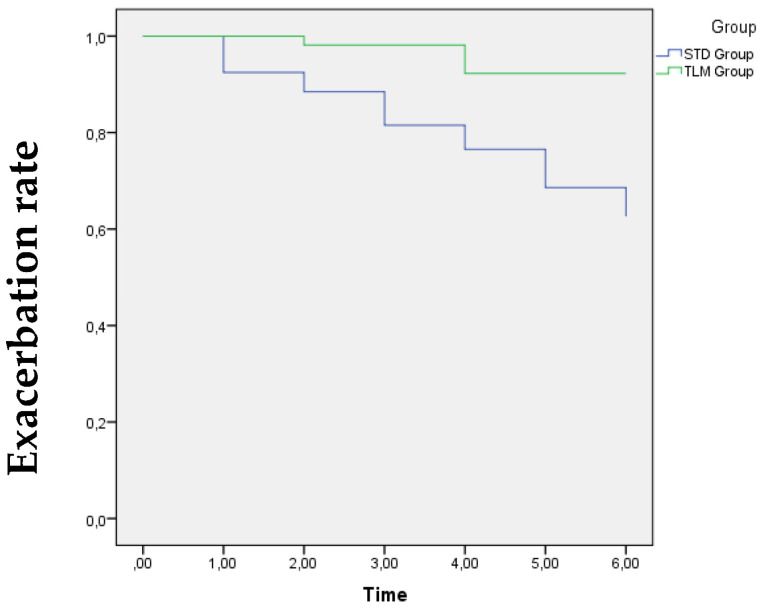
Time to first exacerbation. Log-Rank test *p* < 0.001. Abbreviations: STD: standard care group (STD); TLM: telemonitoring group.

Time to first hospital admission caused by AECOPD was also longer in the TLM group compared to the STD group (2.2 ± 0.5 months vs. 1.5 ± 0.8 months, *p* = 0.04) (Figure 3).

## 4. Discussion

Our study demonstrated a five-fold reduction of ED visits for AECOPD in the TLM group compared to the STD group, along with a reduction of the rate for hospitalization for AECPD. Moreover, the all-cause death rate was also lower among patients receiving home telemonitoring after a 6-month follow-up. The exacerbation free interval was also longer among the TLM group compared to the STD group. COPD causes a substantial challenge to public health, markedly impairing patients’ quality of life and imposing a considerable economic burden. Exacerbation is the most significant problem in managing COPD and stands as a turnover of patients’ quality of life and their respiratory function. Over half of COPD patients are re-hospitalized within 90 days of hospital discharge [9], and each subsequent hospitalization can elevate the risk of further exacerbations, which in turn increases the likelihood of additional rehospitalizations [10]. Many socio-economic, environmental, and medical factors are associated with a higher risk of exacerbations [5,6]. So, several management strategies have been explored; however, prevention is still the most effective approach. Therapeutic adherence is a key component of this prevention strategy. Among the strategies implemented to enhance therapeutic adherence in patients with COPD, therapeutic education is particularly notable [7,8,11,12,13,14,15,16]. Consequently, numerous countries have established COPD self-management programs designed to enhance patients’ skills in recognizing and managing exacerbations. These programs have proven effective in mitigating the progression of COPD on a global scale [7,17]. Coronavirus disease 2019 (COVID-19) restrictions, with the imposition of social distancing, have fueled the adoption of telemedicine as an alternative way to connect patients with their healthcare providers in an efficient and safe way. As a result, the use of virtual medicine has been empowered by the World Health Organization (WHO) declaration on 11 March 2020, leading to a novel reorganization of this healthcare system [18]. Telemedicine employs a variety of technological tools, such as digital platforms and virtual consultations, to facilitate ongoing communication between patients and healthcare professionals [4]. This approach enables frequent reminders about therapeutic regimens and encourages adherence to prescribed treatments. By fostering proactive communication, telemedicine allows healthcare providers to offer timely guidance on treatment strategies and their correct application [6]. For instance, regular reminders about proper inhaler techniques and disease management can enhance symptom control and reduce the frequency of exacerbations among patients with COPD and hospitalizations [19]. Furthermore, telemedicine facilitates close monitoring of respiratory parameters and aids in the early detection of exacerbations, enabling timely intervention before hospitalization becomes necessary [13]. Artificial intelligence and wearable devices are emerging and finding their way into the health sector and may provide new opportunities for early prediction and prevention of COPD exacerbation [20]. This approach involves an ongoing process of instruction and learning tailored to patients’ needs. Traditionally, face-to-face sessions have been time-consuming, but telemedicine now provides a viable option. This modern technology not only improves accessibility and convenience, but it also has the potential to greatly improve patient outcomes when controlling COPD [4].

Moreover, the delayed initiation of treatment at the onset of exacerbation is known to increase the likelihood of hospitalization; raising patient awareness about self-initiating treatment or using telemedicine to quickly contact a physician and start treatment early significantly contributes to reducing hospitalizations. In fact, this study demonstrated a notable reduction in the number of exacerbations following six months of regular therapeutic education via home phone-based telemonitoring. These findings are consistent with the existing literature [11,15]. Several studies have demonstrated the positive effect of telemonitoring on the cumulative hospitalization rate for patients with COPD [8,9,10,19,21]. M. Vitacca et al. have similarly reported a significant decrease in exacerbation rates among patients with COPD receiving therapeutic education through telemonitoring, with fewer hospitalizations and a higher probability of avoiding hospitalization than the control group [17]. Zafar et al. reported that implementing this therapeutic education strategy decreased 30-day all-cause readmissions from 22.7% to 14.7% [22]. Another factor influencing hospitalization rates is rural residency and the limited availability of nearby healthcare facilities, where telemonitoring proves particularly valuable [15]. Telehealth/telemedicine showed positive effects on patients with COPD after hospitalization for an exacerbation, playing a central role in self-management [21]. This approach not only enhances patients’ quality of life but also reduces healthcare costs [10,21,22]. Telemedicine currently represents an innovative approach with a substantial impact on the management of patients with COPD. Integrating telemedicine into patient education offers significant advantages, including enhanced accessibility, remote monitoring, personalized care, and savings in both time and cost [23]. Additionally, it improves treatment adherence and fosters greater patient autonomy. This approach allows for more precise therapeutic adjustments in line with COPD guidelines, leading to improved prognostic outcomes with fewer exacerbations and hospitalizations. Furthermore, patients are more likely to adhere to COPD management recommendations when they have convenient access to educational resources tailored to their specific needs [23]. Concerning mortality rates, a recent 12-month RCT by Kessler, R. et al. revealed a reduced acute care hospitalization days and mortality rate in severe patients with COPD [24]. However, there is still debate concerning telemonitoring and COPD because of a lack of compelling evidence. This could be explained by the heterogenous designs of the various telemonitoring interventions that have been studied to date [25,26,27]. For instance, McDowell, J.E. et al. have not found that telemedicine had a favorable effect on COPD exacerbations. This lack of positive outcome could be due to the fact that telemedicine was employed only for monitoring and early detection of exacerbation symptoms, with no associated interventions or therapeutic instruction. Telemedicine’s potential benefits in minimizing exacerbations may be underutilized unless additional management strategies and educational components are integrated [19]. This study is one of the first to evaluate telehealth’s impact on patients with COPD in North Africa. The educational protocol employed was thorough, encompassing various aspects of the disease, from pathophysiology to management and complications. However, there are certain limitations to acknowledge. First, the relatively short duration of the intervention and a longer duration might provide more substantial insights. Second, this study did not measure or discuss potential oximetry and spirometry changes among participants, and we only conduct phone calls to monitor patients, thus preventing determination of the independent effects of these factors. Third, individuals with advanced underlying conditions were not included in this study, and a possible selection bias would lie in the fact that the patients included must have the aptitude to undergo phone monitoring. In addition, since TM was an active non-pharmacological treatment, neither patients nor the investigator who performed phone calls could be blinded to treatment allocation [28]. Finally, barriers to TLM, including affordability, acceptability, long-term adherence, and cost of the technology, could limit the widespread adoption of the TLM approach [29]. Therefore, the results of this study could not be generalized to the entire COPD population.

## 5. Conclusions

This study emphasizes the role of educational procedures as a vital link in the therapeutic chain for patients with COPD. The incorporation of telemedicine into this procedure contributes to more effective disease management and improved quality of life for patients.

## Figures and Tables

**Table 1:** Baseline characteristics of the groups: standard group (STD, n = 106) and telemonitoring group (TLM, n = 57).

Baseline Characteristics	Total (n = 163)	STD Group (n = 106)	TLM Group (n = 57)	*p*
**Age, (years ± SD)**	66.5 ± 12.4	67.3 ± 12.7	65.043 ± 11.8	0.27
				0.01
Male	112 (68.7)	66 (58.9)	46 (41.4)	
**Employment sector**				0.49
Healthcare sector	4 (2.5)	4 (3.8)	0 (0.0)	
Education sector	7 (4.3)	4 (3.8)	3 (5.3)	
An employee of the state	40 (24.5)	22 (20.8)	18 (31.6)	
Student	1 (0.6)	1 (0.9)	0 (0.0)	
Others	111 (68.1)	75 (70.8)	36 (63.2)	
**Education level**				0.06
Illiterate	36 (22.1)	24 (22.6)	12 (21.1)	
Primary school	56 (34.4)	30 (28.3)	26 (45.6)	
Secondary school	44 (27.0)	28 (26.4)	16 (28.1)	
University	27 (16.6)	24 (22.6)	3 (5.3)	
**Geographical origin**				0.86
Rural area	59 (36.2)	39(36.8)	20 (35.1)	
Urban area	104 (63.8)	67(63.2)	37 (64.9)	
**Distance from home to hospital(km)**				0.06
**≤10**	107 (65.6)	64 (60.4)	43 (75.4)	
**>10**	56 (34.4)	42 (9.6)	14 (24.6)	
PA **Past medical history**				
Diabetes	82 (50.3)	51 (48.1)	31 (54.4)	0.445
Hypertension	103 (63.2)	67 (63.2)	36 (63.2)	0.995
Dyslipidemia	48 (29.4)	27 (25.5)	21 (36.8)	0.129
AF	27 (16.6)	17 (16.0)	10 (17.5)	0.805
Heart failure	38 (23.3)	31 (29.2)	7 (12.3)	0.015
Lung cancer	1 (0.6)	0 (0.0)	1 (1.8)	0.350
Chronic respiratory failure	23 (14.1)	22 (20.8)	1 (1.8)	0.001
**mMRC**				0.059
1	53 (32.5)	42 (39.6)	11 (19.3)	
2	49 (30.1)	29 (27.4)	20 (35.1)	
3	57 (35.0)	32 (30.2)	25 (43.9)	
4	4 (2.5)	3 (2.8)	1 (1.8)	

Abbreviations: AF: atrial fibrillation; mMRC: modified medical research council; SD: standard deviation; STD: standard care group (STD); TLM: telemonitoring group. Quantitative and categorical data were mean ± standard deviation and number (%), respectively.

**Table 2:** Secondary outcomes.

Secondary Outcomes, n (%)	STD Group (n = 106)	TLM Group (n = 57)	OR (95% CI)	*p*
Hospitalizations for AECOPD	47 (44.3)	9 (15.8)	0.23 (0.10–0.52)	<0.001
All-cause death	6 (5.7)	1 (1.8)	0.29 (0.03–2.53)	0.001

Abbreviations: CI = confidence interval; OR  =  odds ratio adjusted by sex; STD: standard care group (STD); TLM: telemonitoring group.

## Data Availability

The data presented in this study are available upon request from the corresponding author (the data are not publicly available due to privacy or ethical restrictions).
